# Poly[tetra­aqua­(μ_8_-butane-1,2,3,4-tetra­carboxyl­ato)distrontium]

**DOI:** 10.1107/S1600536811046265

**Published:** 2011-11-05

**Authors:** Pei-Chi Cheng, Jun-Xiang Zhan, Cheng-You Wu, Chia-Her Lin

**Affiliations:** a Department of Chemistry, R&D Center for Membrane Technology, Center for Nanotechnology, Chung-Yuan Christian University, Chung-Li 320, Taiwan; b Department of Chemistry, Chung-Yuan Christian University, Chung-Li 320, Taiwan

## Abstract

In the title compound, [Sr_2_(C_8_H_6_O_8_)(H_2_O)_4_)]_*n*_, the Sr^II^ ion is coordinated by six O atoms of four symmetry-related ligands and two water mol­ecules in a distorted bicapped trigonal–prismatic environment. The butane-1,2,3,4-tetra­carboxyl­ate ligands lie on inversion centers and bridge Sr^II^ ions, forming a three-dimensional network. Within the three-dimensional structure, there are O—H⋯O hydrogen bonds involving the water mol­ecules and carboxyl­ate O atoms.

## Related literature

For general background to coordination polymers, see: Jiang & Xu (2011 ▶); Kam *et al.* (2007 ▶); Kitagawa *et al.* (2004 ▶); Liu *et al.* (2009 ▶). For related structures, see: Ma & Yan (2009 ▶); Wu (2009 ▶).
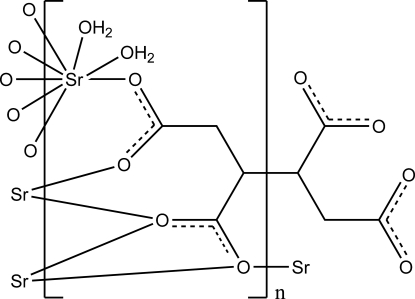


## Experimental

### 

#### Crystal data

[Sr_2_(C_8_H_6_O_8_)(H_2_O)_4_)]
*M* *_r_* = 477.44
Monoclinic, 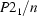

*a* = 8.7085 (4) Å
*b* = 7.9671 (4) Å
*c* = 10.0697 (4) Å
β = 95.409 (2)°
*V* = 695.54 (5) Å^3^
*Z* = 2
Mo *K*α radiation
μ = 7.73 mm^−1^
*T* = 296 K
0.25 × 0.15 × 0.13 mm

#### Data collection

Bruker APEXII CCD diffractometer
Absorption correction: multi-scan (*SADABS*; Bruker, 2010 ▶) *T*_min_ = 0.248, *T*_max_ = 0.433
7782 measured reflections
1746 independent reflections
1443 reflections with *I* > 2σ(*I*)
*R*_int_ = 0.072

#### Refinement

*R*[*F* ^2^ > 2σ(*F* ^2^)] = 0.028
*wR*(*F* ^2^) = 0.061
*S* = 0.97
1746 reflections
100 parameters
H-atom parameters constrained
Δρ_max_ = 0.58 e Å^−3^
Δρ_min_ = −0.46 e Å^−3^

### 

Data collection: *APEX2* (Bruker, 2010 ▶); cell refinement: *SAINT* (Bruker, 2010 ▶); data reduction: *SAINT*; program(s) used to solve structure: *SHELXS97* (Sheldrick, 2008 ▶); program(s) used to refine structure: *SHELXL97* (Sheldrick, 2008 ▶); molecular graphics: *DIAMOND* (Brandenburg, 2010 ▶); software used to prepare material for publication: *SHELXTL* (Sheldrick, 2008 ▶).

## Supplementary Material

Crystal structure: contains datablock(s) I, global. DOI: 10.1107/S1600536811046265/lh5368sup1.cif

Structure factors: contains datablock(s) I. DOI: 10.1107/S1600536811046265/lh5368Isup2.hkl

Additional supplementary materials:  crystallographic information; 3D view; checkCIF report

## Figures and Tables

**Table 1:** Hydrogen-bond geometry (Å, °)

*D*—H⋯*A*	*D*—H	H⋯*A*	*D*⋯*A*	*D*—H⋯*A*
O1*W*—H1*WA*⋯O3^i^	0.85	1.90	2.729 (3)	164.5
O1*W*—H1*WB*⋯O4^ii^	0.85	2.14	2.944 (3)	159.1
O2*W*—H2*WA*⋯O4	0.85	2.06	2.841 (3)	153.1
O2*W*—H2*WB*⋯O1*W*^iii^	0.88	1.99	2.864 (3)	170.1

Symmetry codes: (i) 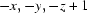; (ii) 
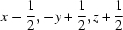
; (iii) 
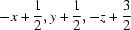
.

**Fig. 1.**
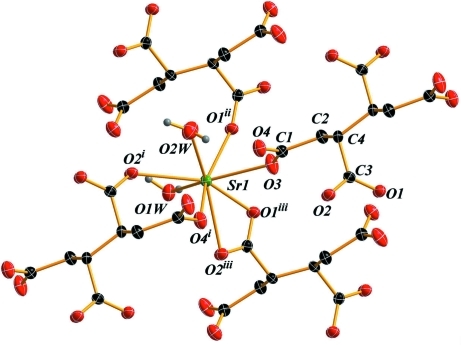
A view of part of the title structure, showing 50% probability displacement ellipsoids. [symmetry codes: (i) 1 - x, -y, 1 - z; (ii) –x + 1/2, y + 1/2, -z + 1/2; (iii) x - 1/2, -y - 1/2, z + 1/2].

**Fig. 2.**
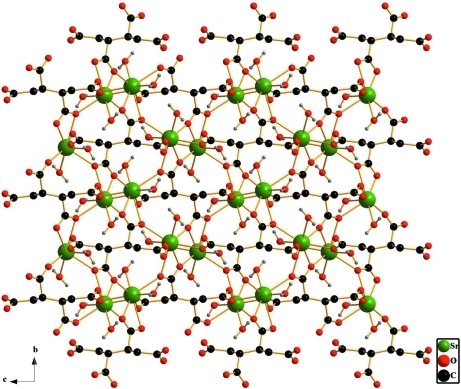
Part of the crystal structure of the title compound viewed along the a axis.

## supplementary crystallographic information

### Comment

Multicarboxylate ligands, are widely used to construct coordination polymers with interesting properties (Kam, *et al.*, 2007; Liu, *et al.*, 2009, Kitagawa *et al.*, 2004; Jiang & Xu, 2011). The butane-1,2,3,4-tetracarboxylato ligand has already been reported in crystal structures  (Ma & Yan, 2009; Wu, 2009). In our continuing investigations on metal coordination polymers we report here the structure of a new Sr coordination polymer based on butane-1,2,3,4-tetracarboxylatic acid.

The asymmetric unit of the title compound consists of one strontium, one half carboxylate ligand and two coordinated water molecules (Fig. 1). The stronium is eight coordinated by six O atoms of four symmetry related ligands and two water molecules. The Sr—O distances range from 2.5491 (18) to 2.688 (2) Å. The ligands bridge the neighboring Sr^II^ centers forming a three-dimensional framework structure (Fig. 2). The hydrogen bonds involving the water molecules and the carboxylate O atoms further stabilize the three dimensional structure.

### Experimental

Solvothermal reactions were carriedout at 363 K for 2 d in a Teflon-lined acid digestion bomb with an internal volume of 23 ml followed by slow cooling at 6 K/h to room temperature. A single-phase product consisting of colorless crystals of was obtained from a mixture of butane-1,2,3,4-tetracarboxylatic acid (C_8_H_10_O_8_, 0.0468 g, 0.2 mmol), Sr(NO_3_)_2_ (0.0847 g, 0.4 mmol), and ethanol(5.0 ml) and H_2_O (1.0 ml).

### Refinement

H atoms were constrained to ideal geometries, with C—H = 0.96-0.98 Å, O—H = 0.85-0.88Å and *U*_iso_(H) = 1.2*U*_eq_(C);*U*_iso_(H) = 1.5*U*_eq_(O).

### Crystal data

**Table:**

[Sr_2_(C_8_H_6_O_8_)(H_2_O)_4_)]	*F*(000) = 468
*M**_r_* = 477.44	*D*_x_ = 2.280 Mg m^−^^3^
Monoclinic, *P*2_1_/*n*	Mo *K*α radiation, λ = 0.71073 Å
Hall symbol: -P 2yn	Cell parameters from 4783 reflections
*a* = 8.7085 (4) Å	θ = 3.0–28.5°
*b* = 7.9671 (4) Å	µ = 7.73 mm^−^^1^
*c* = 10.0697 (4) Å	*T* = 296 K
β = 95.409 (2)°	Tabular, colourless
*V* = 695.54 (5) Å^3^	0.25 × 0.15 × 0.13 mm
*Z* = 2	

### Data collection

**Table:**

Bruker APEXII CCD diffractometer	1746 independent reflections
Radiation source: fine-focus sealed tube	1443 reflections with *I* > 2σ(*I*)
graphite	*R*_int_ = 0.072
Detector resolution: 8.3333 pixels mm^-1^	θ_max_ = 28.7°, θ_min_ = 3.0°
φ and ω scans	*h* = −11→11
Absorption correction: multi-scan (*SADABS*; Bruker, 2010)	*k* = −8→10
*T*_min_ = 0.248, *T*_max_ = 0.433	*l* = −13→13
7782 measured reflections	

### Refinement

**Table:**

Refinement on *F*^2^	Primary atom site location: structure-invariant direct methods
Least-squares matrix: full	Secondary atom site location: difference Fourier map
*R*[*F*^2^ > 2σ(*F*^2^)] = 0.028	Hydrogen site location: inferred from neighbouring sites
*wR*(*F*^2^) = 0.061	H-atom parameters constrained
*S* = 0.97	*w* = 1/[σ^2^(*F*_o_^2^) + (0.0282*P*)^2^] where *P* = (*F*_o_^2^ + 2*F*_c_^2^)/3
1746 reflections	(Δ/σ)_max_ = 0.001
100 parameters	Δρ_max_ = 0.58 e Å^−^^3^
0 restraints	Δρ_min_ = −0.46 e Å^−^^3^

### Special details

**Table:**

Geometry. All e.s.d.'s (except the e.s.d. in the dihedral angle between two l.s. planes) are estimated using the full covariance matrix. The cell e.s.d.'s are taken into account individually in the estimation of e.s.d.'s in distances, angles and torsion angles; correlations between e.s.d.'s in cell parameters are only used when they are defined by crystal symmetry. An approximate (isotropic) treatment of cell e.s.d.'s is used for estimating e.s.d.'s involving l.s. planes.
Refinement. Refinement of *F*^2^ against ALL reflections. The weighted *R*-factor *wR* and goodness of fit *S* are based on *F*^2^, conventional *R*-factors *R* are based on *F*, with *F* set to zero for negative *F*^2^. The threshold expression of *F*^2^ > σ(*F*^2^) is used only for calculating *R*-factors(gt) *etc*. and is not relevant to the choice of reflections for refinement. *R*-factors based on *F*^2^ are statistically about twice as large as those based on *F*, and *R*- factors based on ALL data will be even larger.

### Fractional atomic coordinates and isotropic or equivalent isotropic displacement parameters (Å^2^)

**Table:**

	*x*	*y*	*z*	*U*_iso_*/*U*_eq_	
Sr1	0.22086 (2)	0.04306 (3)	0.60530 (2)	0.01806 (9)	
O3	0.2772 (2)	−0.0137 (3)	0.36082 (19)	0.0333 (5)	
O1W	0.0092 (2)	0.0561 (3)	0.7699 (2)	0.0285 (5)	
H1WA	−0.0857	0.0434	0.7437	0.043*	
H1WB	0.0108	0.1483	0.8122	0.043*	
O2W	0.4040 (2)	0.2833 (3)	0.5536 (2)	0.0405 (6)	
H2WA	0.4624	0.2227	0.5104	0.061*	
H2WB	0.4420	0.3623	0.6089	0.061*	
O1	0.5067 (2)	−0.3138 (2)	0.03110 (18)	0.0249 (4)	
O2	0.69087 (19)	−0.2208 (3)	0.17764 (18)	0.0249 (4)	
O4	0.5123 (2)	0.0870 (3)	0.34605 (19)	0.0281 (5)	
C1	0.3872 (3)	0.0252 (4)	0.2949 (2)	0.0194 (5)	
C2	0.3645 (3)	0.0043 (4)	0.1447 (2)	0.0202 (6)	
H2A	0.3107	0.1013	0.1075	0.024*	
H2B	0.3001	−0.0920	0.1243	0.024*	
C3	0.5739 (3)	−0.1966 (4)	0.0963 (2)	0.0183 (5)	
C4	0.5134 (3)	−0.0185 (4)	0.0755 (2)	0.0174 (5)	
H4A	0.5914	0.0597	0.1156	0.021*	

### Atomic displacement parameters (Å^2^)

**Table:**

	*U*^11^	*U*^22^	*U*^33^	*U*^12^	*U*^13^	*U*^23^
Sr1	0.01577 (12)	0.01900 (17)	0.01883 (12)	−0.00105 (10)	−0.00146 (8)	−0.00014 (10)
O3	0.0215 (9)	0.0593 (17)	0.0201 (9)	−0.0077 (9)	0.0072 (8)	−0.0053 (9)
O1W	0.0222 (9)	0.0352 (14)	0.0281 (10)	0.0005 (8)	0.0025 (8)	−0.0077 (9)
O2W	0.0443 (12)	0.0363 (15)	0.0418 (12)	−0.0080 (11)	0.0088 (10)	−0.0065 (11)
O1	0.0272 (9)	0.0188 (12)	0.0268 (9)	−0.0009 (8)	−0.0074 (8)	−0.0012 (8)
O2	0.0249 (9)	0.0185 (11)	0.0295 (10)	0.0031 (8)	−0.0080 (8)	−0.0007 (8)
O4	0.0196 (9)	0.0405 (15)	0.0237 (9)	−0.0016 (8)	−0.0004 (7)	−0.0038 (9)
C1	0.0171 (11)	0.0219 (16)	0.0193 (12)	0.0025 (11)	0.0027 (9)	0.0002 (11)
C2	0.0157 (11)	0.0272 (17)	0.0177 (11)	−0.0007 (10)	0.0016 (9)	0.0003 (11)
C3	0.0181 (11)	0.0195 (16)	0.0176 (11)	−0.0007 (10)	0.0029 (9)	0.0026 (10)
C4	0.0182 (11)	0.0195 (16)	0.0144 (11)	−0.0016 (10)	0.0016 (9)	−0.0024 (10)

### Geometric parameters (Å, °)

**Table:**

Sr1—O4^i^	2.5491 (18)	O1—Sr1^iv^	2.5689 (17)
Sr1—O1^ii^	2.5690 (17)	O1—Sr1^v^	2.6666 (18)
Sr1—O2W	2.576 (2)	O2—C3	1.260 (3)
Sr1—O1W	2.5946 (19)	O2—Sr1^i^	2.6565 (18)
Sr1—O3	2.5948 (19)	O2—Sr1^v^	2.688 (2)
Sr1—O2^i^	2.6565 (18)	O4—C1	1.260 (3)
Sr1—O1^iii^	2.6666 (18)	O4—Sr1^i^	2.5492 (18)
Sr1—O2^iii^	2.688 (2)	C1—C2	1.516 (3)
Sr1—C3^iii^	3.040 (3)	C2—C4	1.540 (3)
Sr1—H2WA	2.7869	C2—H2A	0.9615
O3—C1	1.254 (3)	C2—H2B	0.9614
O1W—H1WA	0.8501	C3—C4	1.521 (4)
O1W—H1WB	0.8483	C3—Sr1^v^	3.040 (3)
O2W—H2WA	0.8499	C4—C4^vi^	1.544 (5)
O2W—H2WB	0.8836	C4—H4A	0.9800
O1—C3	1.254 (3)		
			
O4^i^—Sr1—O1^ii^	157.86 (6)	O4^i^—Sr1—H2WA	64.6
O4^i^—Sr1—O2W	76.72 (7)	O1^ii^—Sr1—H2WA	99.1
O1^ii^—Sr1—O2W	91.33 (6)	O2W—Sr1—H2WA	17.7
O4^i^—Sr1—O1W	125.67 (6)	O1W—Sr1—H2WA	143.4
O1^ii^—Sr1—O1W	76.42 (6)	O3—Sr1—H2WA	63.1
O2W—Sr1—O1W	126.16 (7)	O2^i^—Sr1—H2WA	80.7
O4^i^—Sr1—O3	82.01 (6)	O1^iii^—Sr1—H2WA	141.8
O1^ii^—Sr1—O3	77.02 (6)	O2^iii^—Sr1—H2WA	132.8
O2W—Sr1—O3	76.20 (7)	C3^iii^—Sr1—H2WA	141.7
O1W—Sr1—O3	145.32 (6)	Sr1^vii^—Sr1—H2WA	125.9
O4^i^—Sr1—O2^i^	82.57 (6)	C1—O3—Sr1	132.92 (17)
O1^ii^—Sr1—O2^i^	110.61 (6)	Sr1—O1W—H1WA	121.7
O2W—Sr1—O2^i^	68.51 (6)	Sr1—O1W—H1WB	112.2
O1W—Sr1—O2^i^	67.72 (6)	H1WA—O1W—H1WB	103.2
O3—Sr1—O2^i^	143.85 (6)	Sr1—O2W—H2WA	95.3
O4^i^—Sr1—O1^iii^	112.11 (6)	Sr1—O2W—H2WB	127.1
O1^ii^—Sr1—O1^iii^	70.75 (7)	H2WA—O2W—H2WB	121.4
O2W—Sr1—O1^iii^	151.93 (6)	C3—O1—Sr1^iv^	155.35 (16)
O1W—Sr1—O1^iii^	71.71 (6)	C3—O1—Sr1^v^	94.78 (14)
O3—Sr1—O1^iii^	78.74 (6)	Sr1^iv^—O1—Sr1^v^	109.25 (7)
O2^i^—Sr1—O1^iii^	137.41 (6)	C3—O2—Sr1^i^	127.36 (17)
O4^i^—Sr1—O2^iii^	70.72 (6)	C3—O2—Sr1^v^	93.61 (16)
O1^ii^—Sr1—O2^iii^	118.62 (5)	Sr1^i^—O2—Sr1^v^	134.76 (7)
O2W—Sr1—O2^iii^	147.38 (6)	C1—O4—Sr1^i^	131.09 (19)
O1W—Sr1—O2^iii^	76.83 (6)	O3—C1—O4	123.7 (2)
O3—Sr1—O2^iii^	97.05 (7)	O3—C1—C2	117.8 (2)
O2^i^—Sr1—O2^iii^	108.31 (3)	O4—C1—C2	118.5 (2)
O1^iii^—Sr1—O2^iii^	48.59 (5)	C1—C2—C4	115.4 (2)
O4^i^—Sr1—C3^iii^	90.58 (7)	C1—C2—H2A	108.3
O1^ii^—Sr1—C3^iii^	94.92 (6)	C4—C2—H2A	108.6
O2W—Sr1—C3^iii^	159.30 (7)	C1—C2—H2B	108.6
O1W—Sr1—C3^iii^	74.52 (6)	C4—C2—H2B	108.0
O3—Sr1—C3^iii^	86.00 (7)	H2A—C2—H2B	107.8
O2^i^—Sr1—C3^iii^	126.65 (6)	O1—C3—O2	122.4 (2)
O1^iii^—Sr1—C3^iii^	24.28 (6)	O1—C3—C4	118.9 (2)
O2^iii^—Sr1—C3^iii^	24.44 (6)	O2—C3—C4	118.7 (2)
O4^i^—Sr1—Sr1^vii^	142.42 (5)	O1—C3—Sr1^v^	60.94 (13)
O1^ii^—Sr1—Sr1^vii^	36.13 (4)	O2—C3—Sr1^v^	61.95 (14)
O2W—Sr1—Sr1^vii^	124.41 (5)	C4—C3—Sr1^v^	172.02 (16)
O1W—Sr1—Sr1^vii^	70.29 (4)	C3—C4—C2	110.1 (2)
O3—Sr1—Sr1^vii^	75.10 (4)	C3—C4—C4^vi^	109.4 (3)
O2^i^—Sr1—Sr1^vii^	132.19 (4)	C2—C4—C4^vi^	111.5 (2)
O1^iii^—Sr1—Sr1^vii^	34.62 (4)	C3—C4—H4A	108.6
O2^iii^—Sr1—Sr1^vii^	82.83 (3)	C2—C4—H4A	108.6
C3^iii^—Sr1—Sr1^vii^	58.82 (5)	C4^vi^—C4—H4A	108.6

Symmetry codes: (i) −*x*+1, −*y*, −*z*+1; (ii) −*x*+1/2, *y*+1/2, −*z*+1/2; (iii) *x*−1/2, −*y*−1/2, *z*+1/2; (iv) −*x*+1/2, *y*−1/2, −*z*+1/2; (v) *x*+1/2, −*y*−1/2, *z*−1/2; (vi) −*x*+1, −*y*, −*z*; (vii) −*x*, −*y*, −*z*+1.

### Hydrogen-bond geometry (Å, °)

**Table:**

*D*—H···*A*	*D*—H	H···*A*	*D*···*A*	*D*—H···*A*
O1W—H1WA···O3^vii^	0.85	1.90	2.729 (3)	164.5
O1W—H1WB···O4^viii^	0.85	2.14	2.944 (3)	159.1
O2W—H2WA···O4	0.85	2.06	2.841 (3)	153.1
O2W—H2WB···O1W^ix^	0.88	1.99	2.864 (3)	170.1

Symmetry codes: (vii) −*x*, −*y*, −*z*+1; (viii) *x*−1/2, −*y*+1/2, *z*+1/2; (ix) −*x*+1/2, *y*+1/2, −*z*+3/2.
